# Surface Characterization of DPPG Films Modified by Chitosan, Hyaluronic Acid and Titanium Dioxide

**DOI:** 10.3390/ijms27083400

**Published:** 2026-04-10

**Authors:** Agata Ładniak, Małgorzata Jurak, Agnieszka E. Wiącek

**Affiliations:** 1 Department of Biomedical and Analytical Chemistry, Institute of Medicine, The John Paul II Catholic University of Lublin, Konstantynów 1J Street, 20-708 Lublin, Poland; 2 Department of Interfacial Phenomena, Faculty of Chemistry, Maria Curie-Skłodowska University, Maria Curie-Skłodowska Square 3, 20-031 Lublin, Poland; malgorzata.jurak@mail.umcs.pl

**Keywords:** mimetic antibacterial membrane, topography, surface free energy, biopolymers, titanium dioxide

## Abstract

This study focused on elucidating the effects of chitosan (Ch), hyaluronic acid (HA), and titanium dioxide nanoparticles (nano-TiO_2_) on the physicochemical characteristics of a model bacterial membrane (layer) composed of the phospholipid DPPG (1,2-dipalmitoyl-*sn*-glycero-3-phospho-*rac*-(1-glycerol) sodium salt). The membrane was prepared on mica using the Langmuir–Blodgett (LB) technique from an aqueous subphase containing Ch, HA and/or TiO_2_. Its surface properties were subsequently characterized by optical profilometry and surface free energy estimation. The nanoscale topography of the DPPG layer provided a biomimetic platform that reflects the organization of bacterial membranes, enabling a precise evaluation of how external agents, such as Ch, HA, and nano-TiO_2_, modify the surface’s structural and energetic properties. The results showed that the LB films exhibit mildly heterogeneous topography, which can be attributed to lipid domains with distinct molecular packing densities. Depending on the type of biopolymer employed with TiO_2_, distinct topographic architectures of the DPPG monolayers were obtained. Furthermore, the presence of nano-TiO_2_ was clearly manifested as a topographic irregularity, while the analysis of hydrophilic–hydrophobic properties revealed a structurally perturbed lipid film. The results provide detailed insight into how these specific molecules (Ch, HA, nano-TiO_2_) interact at the molecular level with model bacterial membranes, offering a comprehensive picture of cell–microenvironment interactions.

## 1. Introduction

Materials combining chitosan, hyaluronic acid, and titanium dioxide (TiO_2_) are gaining significant attention due to their potential synergistic antibacterial properties and biocompatibility. These composites are widely explored for biomedical applications such as wound dressings, implants, and drug delivery systems [1,2,3,4,5,6].

Chitosan (Ch) is a natural polysaccharide derived from chitin, characterized by its positive charge (cationic nature) in acidic environments. The primary antibacterial mechanism of chitosan involves electrostatic interactions between its positively charged amino groups and the negatively charged bacterial cell membranes [2,7]. Chitosan-based materials have been shown to enhance antibacterial properties when combined with other agents like metal oxides [8]. For instance, chitosan/TiO_2_ nanocomposites demonstrated strong antibacterial activity against *Xanthomonas oryzae pv. oryzae*, outperforming the individual components [9].

Hyaluronic acid (HA) is a naturally occurring glycosaminoglycan known for its biocompatibility, hydrophilicity, and role in tissue regeneration. While HA itself has limited direct antibacterial properties, it enhances wound healing and modulates inflammation, creating an environment less conductive to bacterial colonization. HA can also serve as a matrix to stabilize or deliver antibacterial agents such as chitosan or TiO_2_ nanoparticles, improving their efficacy [3]. When combined with chitosan, HA improves material flexibility, moisture retention, and biocompatibility, which supports tissue regeneration while maintaining antibacterial activity [3,4].

Titanium dioxide (TiO_2_) is chemically stable, non-toxic, and economical. TiO_2_ is known for its photocatalytic properties, which can be harnessed for antibacterial applications. Modifications such as doping with metals or coupling with other materials (e.g., chitosan) can extend its activity into the visible light range. When combined with chitosan, TiO_2_ enhances the antibacterial efficacy of the composite material [4,5,6,9], e.g., against foodborne pathogens like *Campylobacter jejuni* and *Listeria monocytogenes* [10].

**Figure 1:**
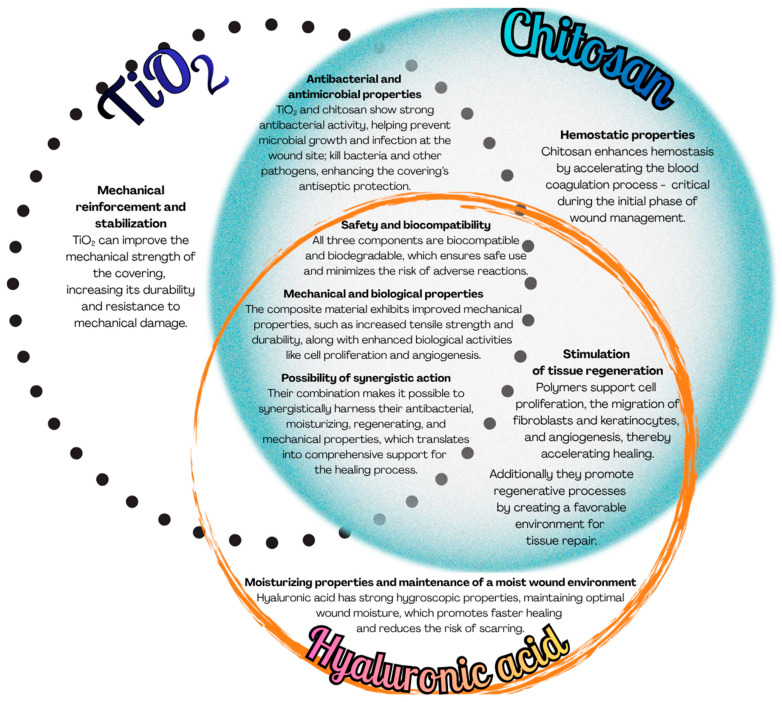
Functional properties of chitosan, hyaluronic acid and titanium dioxide system.

A composite system comprising chitosan, titanium dioxide, and hyaluronic acid integrates the distinct physicochemical and biological properties of these three constituents as summarized in Figure 1, thereby creating a highly promising material for, among other applications, the development of advanced wound dressings with improved healing properties.

To better understand the molecular mechanisms underlying the antibacterial performance of such materials, model systems that mimic biological membranes are essential. The Langmuir monolayer technique offers a well-established model for such investigations under controlled physicochemical conditions. In this context, the study of surface properties of 1,2-dipalmitoyl-*sn*-glycero-3-phospho-*rac*-(1-glycerol) sodium salt (DPPG) films modified by chitosan, hyaluronic acid, and titanium dioxide provides valuable insights into how these components interact with the negatively charged phospholipid as a representative of a class typical of bacterial membranes. Moreover, Langmuir films, which are monomolecular layers formed at the air–water interface, can be transferred onto solid substrates (such as mica sheets or glass plates) to create Langmuir–Blodgett (LB) films [11]. These films have been extensively studied for their unique properties and potential applications in various fields. Surface roughness is a key physicochemical parameter that exerts a pronounced influence on the interaction between microorganisms and material interfaces. Even nanoscale variations in topographical features can modify the spatial distribution of electrostatic charges and, hence, surface free energy and wettability, thereby regulating bacterial adhesion and subsequent biofilm development. Consequently, precise control of the roughness of Langmuir–Blodgett films is crucial for optimizing their biological performance, particularly in applications involving antimicrobial coatings, biosensing platforms, and biomaterials engineering. Belonging to the phosphatidylglycerol class, DPPG serves as an appropriate model for Gram-negative and Gram-positive bacterial membranes, where electrostatic interactions play a crucial role in determining cell adhesion, permeability, and susceptibility to antimicrobial agents. Its behavior in Langmuir monolayers and Langmuir–Blodgett (LB) films reflects key aspects of the organization and stability of biological membranes.

In this study, we conducted a comprehensive topography and hydrophilic–hydrophobic character investigation of phospholipid DPPG monolayers transferred onto mica sheets via the Langmuir–Blodgett technique. Before deposition, DPPG Langmuir monolayers spread on subphases containing chitosan, hyaluronic acid, and titanium dioxide were compressed to 35 mN/m to mimic the molecular packing density characteristic of natural bacterial membranes. The topography characterization and analysis of hydrophilic–hydrophobic properties of DPPG LB films provide a basis for correlating their surface structure with adhesive properties toward bacteria, as well as for understanding the mechanisms of action of biopolymers and nanoparticles in the context of biomimetic membranes.

## 2. Results

### 2.1. Topographical Analysis

**Figure 2:**
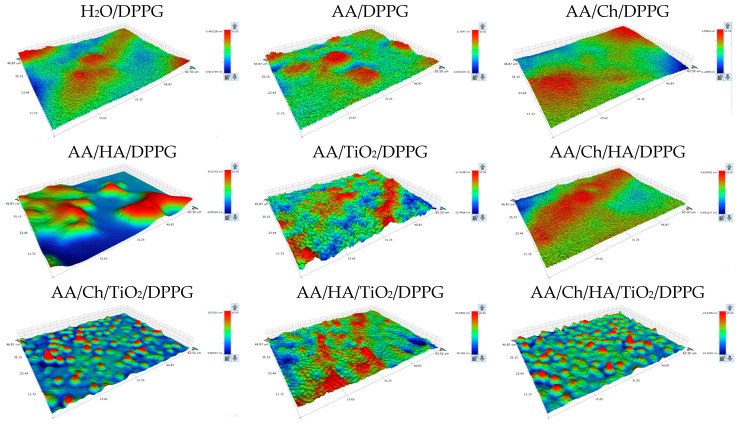
Profilometric images depicting the surface topography of DPPG monolayers transferred onto mica sheets via the Langmuir–Blodgett deposition technique from individual (single- or multi-component) subphases.

**Figure 3:**
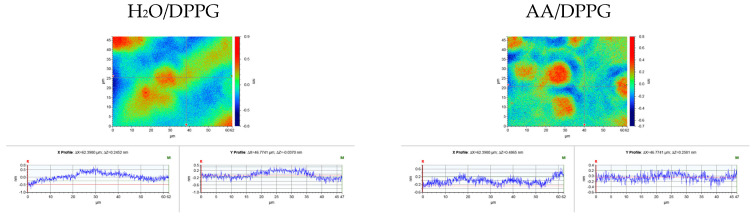

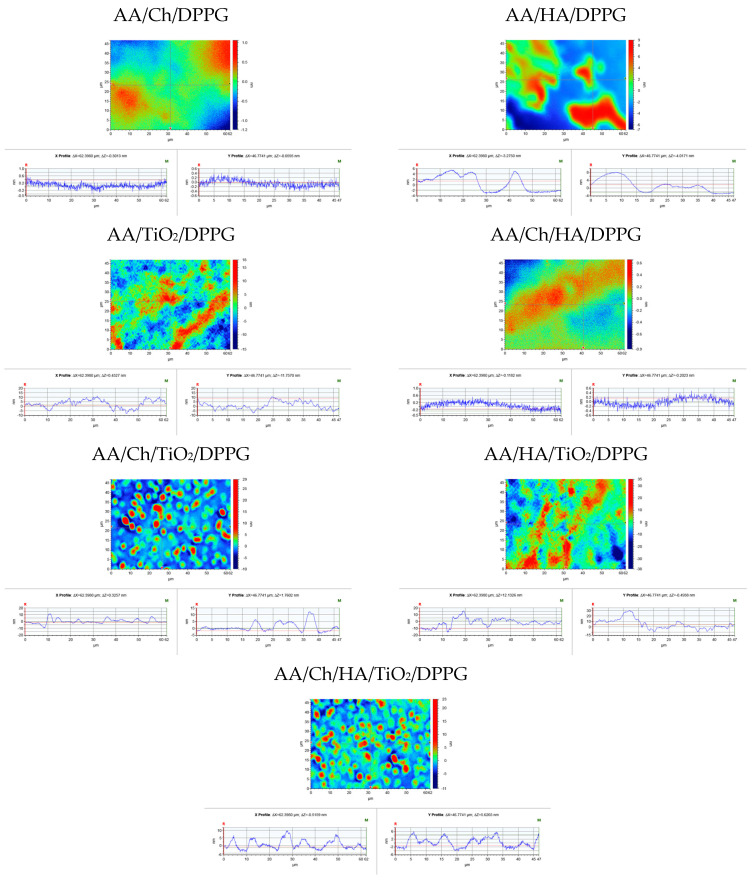
XY profiles along the lines marked on the maps, demonstrating the surface irregularities of DPPG monolayers transferred onto mica sheets via the Langmuir–Blodgett deposition technique from individual (single- or multi-component) subphases.

While the thin films of Ch, HA, and TiO_2_ were characterized in a previous study [12], the current experiments focus on the properties of DPPG monolayers modified by these additives. The surface topography and height profiles of the transferred films, obtained via optical profilometry, are presented in Figure 2 and Figure 3.

To characterize surface roughness, the parameters *R_a_*, *R_q_*, and *R_t_* (Table 1) were employed. These standardized topographic (profile) parameters are routinely used for the quantitative assessment of surface texture. *R_a_* (arithmetical mean deviation) represents the most widely adopted general indicator of roughness; *R_q_* (root mean square roughness) exhibits higher sensitivity to pronounced irregularities; and *R_t_* (total height of the profile) denotes the maximum vertical distance between the highest peak and the lowest valley within the entire evaluation length. All obtained values were in the order of nanometers.

Analyzing the obtained profilometer images, it can be concluded that the films exhibit a mildly heterogeneous morphology. As stated in a previous paper [13], this heterogeneity can be attributed to the presence of lipid domains with distinct molecular packing densities. Furthermore, regions displaying variations in optical reflectivity are observed, which are associated with differences in local film thickness or the orientation of specific segments within the lipid layer [14].

By comparing the optical profilometry images of DPPG monolayers transferred onto mica from individual solutions/dispersions and their mixtures (Figure 2) with corresponding images of bare mica surfaces in contact with phospholipid-free subphases [12], it can be inferred that a fraction of the subphase constituents underlying the DPPG interface is co-transferred onto the mica substrate along with the monolayer.

In general, the presence of a DPPG monolayer on the mica surface contributed to a slight smoothing of most examined surfaces compared to mica coated with phospholipid-free subphase films (Figure 3). This smoothing occurred while maintaining the correlation in the variability of the individual parameters, i.e., *R_a_*, *R_q_*, *R_t_*. The exceptions were samples prepared using AA/HA, AA/Ch/TiO_2_, and AA/Ch/HA/TiO_2_, where higher topographical parameter values were recorded compared to the surfaces without DPPG. This observation suggests that lipid molecules accumulated within the surface depressions, thereby decreasing the structural roughness parameters (*R_a_*, *R_q_*) and rendering the surface topography effectively more planar at the nanometric scale. Alternatively, this may be attributed to a disturbance of the lipid film structure rather than the topography itself. If the film exhibits structural imperfections, such as defects, voids, or domains with heterogeneous mass density, it indicates a lack of long-range crystalline order. Nonetheless, its presence as a continuous thin lipid layer is sufficient to smooth and effectively planarize the underlying substrate. Consequently, the film creates a new, chemically more homogeneous and hydrophobic surface, even though these structural defects prevent it from being an ideal, uniform LB layer.

Smoothing of the substrate surface following Langmuir–Blodgett lipid film deposition, even when the film structure is disturbed, may result from surface uniformity, specific molecular interactions, and the properties of the characterization techniques. Previous research by other authors also confirms that the LB process enables the formation of homogeneous layers capable of smoothing the substrate surface [15,16,17,18,19,20]. In turn, for DPPG films transferred from the AA/HA, AA/Ch/TiO_2_, and AA/Ch/HA/TiO_2_ subphases, an increase in the values of the topographical parameters was observed compared to surfaces without phospholipid. This increase is attributed to specific interactions between the components of the supporting subphase (i.e., HA, Ch, TiO_2_) and the DPPG molecules.

Regarding the surface topography of the examined samples (Figure 2), distinct differences were observed depending on the composition of the subphase from which the DPPG monolayers were transferred. Relatively smooth DPPG films with slight undulations were obtained from the biopolymer-containing subphases. For most samples (Table 1), the *R_t_* parameter did not exceed 2.3 nm. These surfaces were characterized by nano-domains exhibiting various degrees of ordering and diverse morphologies, for example, circular for mica/AA/Ch/DPPG or band-like for mica/AA/Ch/HA/DPPG. These structures result from overlapping of electrostatic interactions between phosphate groups and hydrophobic interactions of the alkyl chains of neighboring DPPG molecules. More pronounced changes were only observed for mica/AA/HA/DPPG, where the *R_t_* parameter reached ~16 nm, manifesting as irregular, streamlined protrusions.

Analysis of the topographic images of the DPPG film surfaces deposited from TiO_2_-containing subphases indicates that the presence of TiO_2_ leads to a pronounced increase in the surface roughness. Specifically, the *R_t_* values ranged from ~33 nm for mica/AA/TiO_2_/DPPG to ~74 nm for mica/AA/HA/TiO_2_/DPPG. Analyzing the distribution of TiO_2_ across the examined surface allows for preliminary conclusions regarding TiO_2_-DPPG and TiO_2_-biopolymer-DPPG interactions. Previous studies have shown that TiO_2_ on mica surfaces, both alone and in combination with biopolymers, is relatively homogeneously distributed [12,13]. However, in the presence of a DPPG monolayer, uniform distribution was only maintained in combination with Ch, i.e., for the mica/AA/Ch/TiO_2_/DPPG and mica/AA/Ch/HA/TiO_2_/DPPG surfaces. Previous analysis [4,21,22] using dynamic light scattering (DLS), the Turbiscan Stability Index (TSI), and electrophoretic mobility measurements (used for zeta potential determination) indicated that the addition of chitosan modifies the physicochemical characteristics of the aqueous titanium(IV) oxide dispersion by affecting the distribution of electrostatic forces between the particles. It was shown that titanium(IV) oxide particles combined with chitosan exhibit enhanced physicochemical properties compared to pure TiO_2_. Moreover, FTIR and XPS spectroscopy [4,22,23] confirm the formation of Ch–TiO_2_ linkage.

In contrast, for the mica/AA/TiO_2_/DPPG and mica/AA/HA/TiO_2_/DPPG surfaces, localized agglomerations of TiO_2_ particles were observed in the form of band-like structures. These formations indicate strong interactions between the positively charged TiO_2_ particles and the negatively charged phospholipid headgroups. Such interactions likely weaken the attractive forces between neighboring DPPG molecules, leading to the observed morphological reorganization.

### 2.2. The Hydrophilic–Hydrophobic Properties Estimation

Both the chemical composition and the surface roughness of a biomaterial affect its wettability. Polar surfaces interact with biological fluids, primarily water [24,25], through specific functional groups such as carboxyl and hydroxyl groups [25,26,27]. The key mechanism in this process is the water–Lewis acid interaction (electron-deficient molecules) or with electron-donor functional groups (−NH_2_, −COOH, −COC−, −OH). Hydrophilic surfaces engage in various intermolecular interactions, including ionic forces, van der Waals forces, electron-donor/acceptor interactions, and oxygen–hydrogen bridges. In contrast, hydrophobic surfaces contain hydrocarbon chains or aromatic compounds with −CH_3_ and −CH_2_− groups and interact with solvent molecules mainly through van der Waals forces, while coordination (donor–acceptor) bonds are virtually absent [24,25,27,28].

**Figure 4:**
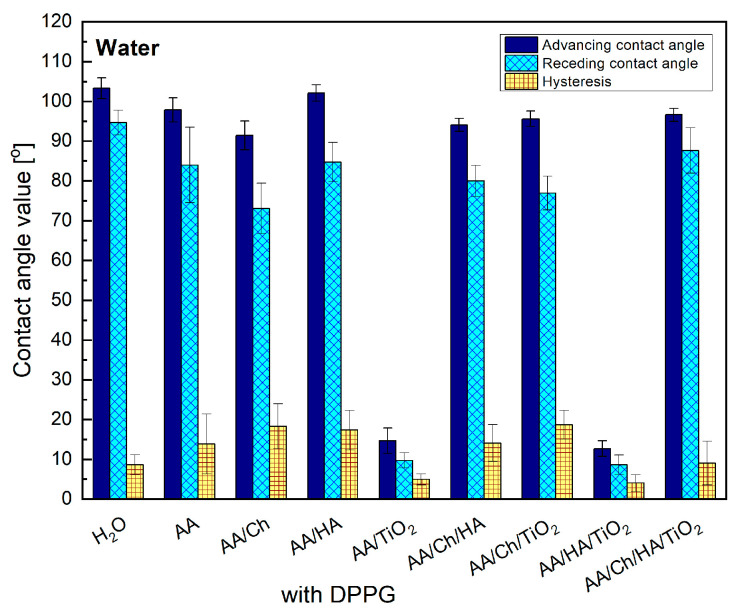
Advancing and receding contact angles of water on the DPPG monolayers transferred onto mica sheets from different subphases containing Ch, HA and/or TiO_2_. Contact angle hysteresis values are also shown.

This study demonstrates that the subphase components (Ch, HA, TiO_2_) significantly change the type and magnitude of these interactions, thereby modifying the wettability of DPPG monolayers (Figure 4).

After transferring the DPPG monolayer from the subphase onto the mica substrate, water contact angles increased markedly for all samples except mica/AA/TiO_2_/DPPG (Figure 4). This trend indicates successful DPPG deposition, with the molecules preferentially oriented such that their hydrocarbon chains are directed toward the air. Subsequent wettability analysis showed reduced surface polarity in all systems.

DPPG monolayers transferred from subphases containing polymers (Ch and/or HA) exhibited slightly lower advancing water contact angle values, accompanied by discernible variations in contact angle hysteresis. These findings suggest a potential reorganization of phospholipid molecules upon interaction with the water droplet, likely resulting from (I) alterations in monolayer packing density that weaken intermolecular interactions between neighboring DPPG molecules and (II) incorporation or influence of subphase components within the DPPG film. In contrast, the extremely low contact angle values measured on mica/AA/TiO_2_/DPPG and mica/AA/HA/TiO_2_/DPPG surfaces indicate pronounced structural perturbations in the DPPG films induced by the presence of TiO_2_.

Given that the wettability of the biomaterial surface is influenced by many factors, including surface chemistry, composition, and topography [29,30], evaluating surface free energy (SFE) has become increasingly common. This approach provides a more precise quantification of the relative magnitudes of polar and nonpolar components [31].

**Figure 5:**
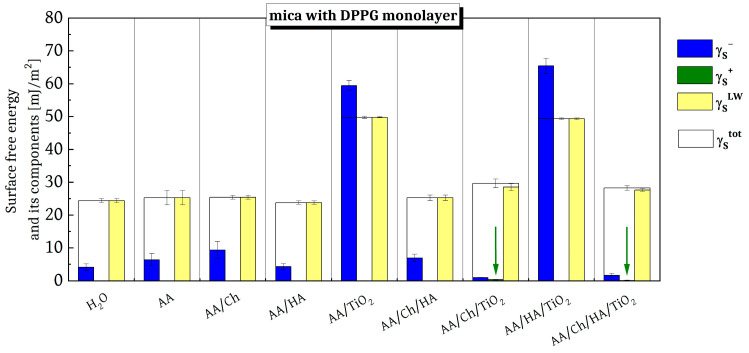
The values of surface free energy and its components obtained for DPPG films transferred onto mica sheets from the tested subphases.

The obtained values of the total SFE ($\gamma_{s}^{tot}$) and its components are presented in Figure 5. Compared to the data for mica surfaces without a phospholipid film [12], a decrease in both $\gamma_{s}^{tot}$ and all of its components and parameters was observed across all examined surfaces. In general, the values of $\gamma_{s}^{tot}$ and $\gamma_{s}^{LW}$ for the individual surfaces ranges from 23 to 30 mJ/m^2^, and these fluctuations can be attributed to subphase components that were co-transferred with the DPPG Langmuir film onto the solid substrate. A disappearance of the electron-acceptor parameter ($\gamma_{s}^{+}$) was also observed; its small contribution was revealed only on the mica/AA/Ch/TiO_2_/DPPG (~0.33 mJ/m^2^) and mica/AA/Ch/HA/TiO_2_/DPPG (~0.06 mJ/m^2^) surfaces, marked with a green arrow. In these two cases, the electron-donor parameter ($\gamma_{s}^{-}$) exhibited the lowest contributions, amounting to 0.99 and 1.64 mJ/m^2^, respectively.

Notably, the values of the components and parameters of the total SFE for mica coated with AA/TiO_2_/DPPG and AA/HA/TiO_2_/DPPG differed significantly from those obtained for the other investigated surfaces. The $\gamma_{s}^{LW}$ values were comparable to those reported for analogous systems without phospholipid, as presented in our previous study [12]. Moreover, these samples showed the highest contribution of the $\gamma_{s}^{-}$ parameter, resulting in the highest $\gamma_{s}^{tot}$ values. This observation suggests that TiO_2_ induces pronounced perturbations in the DPPG monolayer structure. Since an increase in the electron-donor parameter typically results in a more hydrophilic surface [32,33,34], these findings imply a disruption of the expected hydrophobicity afforded by the oriented DPPG molecules.

Furthermore, SFE calculations performed using the Lifshitz–van der Waals/acid–base (LWAB) approach indicated the presence of steric stabilization between Ch and TiO_2_. Specifically, the chitosan layer formed on the TiO_2_ surface increases the separation between TiO_2_ particles and diminishes their electrostatic interactions, thereby reducing the tendency to aggregate. In contrast, the HA-TiO_2_ combination forms a polymer network in which TiO_2_ nanoparticles are dispersed such that they remain in contact with the external environment. This relationship between the interactions and the behavior of the biopolymer-TiO_2_ system is consistent with our previous findings [12,13,35,36].

Moreover, chitosan, HA, and TiO_2_ have been demonstrated to significantly affect the properties of lipid films transferred onto a solid substrate. This was evidenced by their interactions with the phospholipid 1,2-dipalmitoyl-*sn*-glycero-3-phosphocholine (DPPC), as described in our previous publications [13,35,36]. Studies have shown that the presence of these components in the subphase modifies the phospholipid monolayer surface, leading to significant differences in the values of the total surface free energy and its components [35]. Detailed considerations regarding changes in the SFE are presented in the next section.

## 3. Discussion

The significance of this kind of study is particularly important from a biological point of view. Phospholipids, which form the lipid bilayer, constitute the fundamental structural component of cell membranes, serving as a barrier that separates the cytoplasm from the external environment. The integrity of this barrier is crucial for cell survival, as it maintains electrochemical gradients, appropriate ion concentrations, and stable physicochemical conditions inside the cell. Simultaneously, the lipid bilayer exhibits a specific fluidity essential for the proper insertion and functioning of membrane proteins, including transporters and enzymes. Disruptions in lipid organization can alter the activity of these proteins, potentially impairing key cellular processes such as nutrient transport, metabolite removal, and energy synthesis. On the other hand, studying the wettability of bacterial membranes, under conditions that reflect the structure and organization of the cell membranes of living microorganisms in the presence of antibacterial substances, represents an important tool for analyzing changes. Wettability parameters, such as contact angle and surface free energy, make it possible to indirectly assess the molecular-level reorganization of membrane components induced by external factors. Furthermore, topography (optical profilometry) is useful for revealing the microscopic effects of these interactions in adsorption processes, membrane reorganization or emerging defects. Consequently, this approach enables the identification of the mechanisms of action for antibacterial substances, particularly those that act directly on the cell membrane structure.

Another aspect of this research involves the lipid bilayer’s role as a selective barrier. By limiting the free passage of most molecules, the bilayer allows cells to maintain internal homeostasis. In this context, wettability studies provide valuable insight into changes in membrane permeability that may result from destabilization, increased surface hydrophilicity, or the formation of structural defects. These insights are applicable not only to bacteria but also to the broader effects of excess ions or toxins.

On the other hand, the molecular arrangement in Langmuir films significantly influences topography, which is shaped by various molecular interactions, surface pressure during formation, and substrate properties. In our previous publications [12,13,35,36], we characterized the surface topography, as well as wettability, surface free energy, work of adhesion of films composed of chitosan, hyaluronic acid, and titanium dioxide (and their mixtures) deposited on glass and mica sheets. This research, a direct continuation of our previous studies, focuses on analogous systems in the context of their antibacterial potential, employing the anionic phospholipid DPPG as a model component of bacterial membranes. The use of DPPG enables the simulation of interactions occurring at the interface between bacterial cell envelopes and biofunctional surfaces containing chitosan, hyaluronic acid, and titanium dioxide. By analyzing the behavior of DPPG films in the presence of these compounds, we aim to elucidate the molecular mechanisms governing membrane perturbation, stability, and potential disruption. These processes are directly related to the antibacterial activity of the studied materials. This approach bridges the gap between physicochemical characterization and biological performance, offering a molecular-level explanation for the observed antibacterial effects of chitosan-, hyaluronic acid-, and TiO_2_-based systems.

Based on our previous research, we know that the surfaces of films containing Ch, HA, and TiO_2_ are characterized by high surface energy, hydrophilicity, and nanometric roughness [12,13]. Changes in topography and wettability significantly affect interactions with biological membranes, which is important for the biocompatibility of materials [12,13,35,36]. To understand how molecular arrangement correlates with topographical features, knowledge of surface pressure-area isotherms is essential. They provide a quantitative measure of how molecular packing changes with surface pressure and offer insights into the molecular organization and interactions within the films [37,38]. Complementary to this, Brewster angle microscopy (BAM) provides a visualization of the micrometric structure and molecular organization of the films [39]. However, Langmuir isotherms describe only the 2D organization of molecules at the air–water interface. Once the monolayer is transferred onto a solid substrate, it becomes possible to directly observe the surface topography and determine the uniformity, roughness, and defects of the film. This approach enables verification of whether the structural features resulting from the isotherms actually correspond to an ordered, stable layer on the support.

An interesting observation was the smoothing of the carrier surface after deposition of the DPPG Langmuir monolayer from the AA/HA, AA/Ch, AA/TiO_2_, and AA/Ch/HA subphases (with respect to the surface without lipid [12]), despite its local structural disturbances [40]. It is widely known that chitosan (Ch) is a cationic polysaccharide containing amino groups (–NH_2_), which become protonated (–NH_3_^+^) in an acidic environment [41,42,43,44]. Conversely, the phospholipid used in this research, DPPG, possesses negatively charged phosphate groups; therefore, chitosan interacts strongly with the membrane surface via electrostatic forces. Ch penetrates the intermolecular spaces between DPPG molecules to interact with these phosphate groups. This interaction promotes a more vertical orientation of the DPPG molecules at the interface, and ultimately, Ch remains incorporated within the monolayer even under conditions of high lateral compression [40,42]. As a result, chitosan inhibits the formation of the highly organized, densely packed monolayer typical of DPPG, which is physicochemically described as a solid state [40]. Moreover, images obtained by BAM suggest that Ch is preferentially localized in loosely packed monolayer regions corresponding to the liquid-expanded phase, thereby generating Ch-enriched and lipid-poor domains [40,42]. This observation indicates that the positively charged Ch may induce a spatial separation of phosphate groups also within the DPPG headgroup region. Beyond electrostatic interactions, chitosan is also capable of establishing hydrophobic interactions with the lipid acyl chains [40,42,43,45]. In addition, when both chitosan and HA are present, they can form complex multilayer structures on DPPG membranes, significantly altering their physicochemical properties. Such processes can be utilized to design drug delivery systems with controlled release and targeted delivery capabilities [46,47,48].

The lipid molecules can fill the pores and micron-scale irregularities of the substrate. This “filler effect” involves the physical occupation of depressions and the subsequent smoothing of the topography, which leads to the formation of a more homogeneous layer, even if it is not an ideally ordered monolayer. Literature reports suggest that TiO_2_ nanoparticles cause significant deformation in lipid bilayers, particularly those composed of anionic lipids like DPPG. This deformation is more pronounced compared to zwitterionic or other anionic lipids [44]. The interaction with TiO_2_ can lead to local softening of the bilayer, but an increase in overall thickness suggests a stiffening effect despite these local perturbations [49,50].

As shown in Table 1, an increase in the values of topographic parameters, relative to the surface without phospholipid [12], occurred for DPPG films transferred from the following subphases: AA/HA, AA/Ch/TiO_2_, and AA/Ch/HA/TiO_2_. This indicates the existence of strong interactions between the subphase components and the DPPG molecules, leading to structural disturbances in the membrane. As a result, the arrangement of phospholipid molecules at the interface changes. Considering the compression isotherm of the DPPG monolayer on the AA/HA subphase, the monolayer elasticity [40], and the XY profiles of profilometric images (Figure 2 and Figure 3) for the mica/AA/HA/DPPG surface, it can be concluded that at the moment of transfer (π = 35 mN/m), local aggregation of DPPG molecules could have occurred. This results in the formation of both lipid-rich and lipid-poor regions.

Previous studies have shown that the area occupied by a single molecule in a tightly packed monolayer was practically identical to that of DPPG on water. Moreover, the elasticity was preserved, suggesting the absence of HA within the tightly packed monolayer [40]. While isotherm measurements provide information about the molecular organization and packing behavior of the monolayer at the air–water interface, they do not fully reflect the structural and energetic characteristics of the films deposited on solid supports, i.e., conditions that more closely represent real biological interactions. However, based on this research, we can confirm that subtle variations in the profile of the π-A isotherms may arise from repulsive interactions between the negatively charged –PO_4_^−^ headgroups of DPPG and the carboxyl groups of HA. In addition, strong intermolecular hydrogen bonding between the glycerol hydroxyl groups and the phosphate moieties of adjacent lipid molecules can dominate, leading to tighter molecular packing and increased monolayer rigidity [44]. Considering that the mica surface covered with the AA/HA film in the absence of DPPG was topographically smooth (*R_t_* = 2.9 nm) [12], it can be concluded that the surface irregularities observed for the mica/AA/HA/DPPG system (Figure 2, Table 1) are generated by the DPPG molecules in conjunction with the HA “sublayer”. These modifications occurred on the nanometer scale and were therefore detectable only in the profilometric images; however, they did not manifest in the energetic parameters (Figure 5), as their magnitude was too small relative to the microliter volumes of the probe liquids used in those measurements.

An analogous analysis of the experimental data was performed for the AA/Ch/TiO_2_ and AA/Ch/HA/TiO_2_ systems. As previously reported, a single lipid molecule in the densely packed DPPG monolayer formed on these subphases, occupies a substantially larger area compared with the other systems examined [40]. Furthermore, these subphase mixtures impeded the formation of a compact and homogeneous DPPG monolayer due to the presence of chitosan and TiO_2_, indicating strong interactions between the subphase components and DPPG molecules throughout the entire compression process. By analyzing the topographical features together with surface free energy parameters, it is possible to correlate the molecular organization observed in the Langmuir isotherms with the actual physicochemical behavior of the films on solid substrates. This correlation allows for a deeper understanding of how modifications in monolayer composition affect the interfacial energy balance, hydrophilic–hydrophobic properties, and potential antibacterial activity. In light of the above, when interpreting topographical profiles (Figure 3) and the energetic parameters (Figure 5) for DPPG films transferred from these subphases onto mica, the observed irregularities can be ascribed to the presence of TiO_2_ located immediately beneath the DPPG layer. The presence of nano-TiO_2_ is clearly manifested as relatively homogeneously distributed topographical protrusions, whereas the analysis of hydrophilic–hydrophobic properties reveals a structurally perturbed lipid film. However, the extent of this perturbation is insufficient to produce significant discrepancies in the overall experimental results.

Surface roughness significantly influences surface free energy. Increased roughness can either enhance or reduce surface free energy depending on the material and treatment. In this case, the presence of nano-TiO_2_ modifies the surface, increasing surface roughness and, in turn, surface free energy due to enhanced wettability. For the mica/DPPG base systems, which are usually hydrophobic, partial to non-wetting behavior with water should be expected, regardless of roughness at the nano [51] or even micro scale [52]. Nevertheless, when the monolayer was perturbed by the presence of other substances that created structural irregularities, the experimental observations differed significantly. A distinct presence of TiO_2_ in the topographic images was also observed for DPPG monolayers transferred onto the mica surface from AA/TiO_2_ and AA/HA/TiO_2_ subphases. In both cases, however, the distribution of TiO_2_ particles was not as uniform as that observed for the mica/AA/Ch/TiO_2_/DPPG and mica/AA/Ch/HA/TiO_2_/DPPG surfaces. Moreover, the texture of these surfaces differed clearly from the morphology of mica coated with analogous AA/TiO_2_ and AA/HA/TiO_2_ films but lacking the DPPG monolayer, as described in our previous paper [12]. It is well established that TiO_2_ nanoparticles exhibit strong interactions with anionic phospholipids such as DPPG, predominantly mediated by electrostatic forces. Additionally, under acidic conditions, where TiO_2_ acquires a net-positive surface charge, its affinity for negatively charged lipid membranes increases, thereby promoting more pronounced membrane destabilization [53,54]. This leads to changes in phase behavior, viscoelastic properties, and the dynamic response of the membranes [40]. Furthermore, TiO_2_, mainly in the form of nanoparticles, generates reactive oxygen species (ROS) upon exposure to light, which can damage bacterial membranes through oxidative stress. The ability of TiO_2_ to disrupt membrane integrity and enhance permeability is a key factor in its antibacterial activity. For example, studies have shown that TiO_2_ can cause severe membrane damage in both Gram-negative (*E. coli*) and Gram-positive (*S. aureus*) bacteria, with ROS production playing a significant role [55]. Our observations indicate that these strong interactions between TiO_2_ and DPPG can lead to the complete disintegration of the DPPG monolayer. Consistent with previous reports [40], the incorporation of TiO_2_ induces a pronounced reduction in the packing density of DPPG monolayers over the entire compression process. This effect can be attributed to a substantial weakening of the intermolecular attractive forces between DPPG molecules. Consequently, the formation of a compact and homogeneous layer is hindered, leaving the DPPG monolayers in a state characterized by decreased packing density and reduced structural order [40].

Furthermore, the topographical changes observed for mica/AA/TiO_2_/DPPG and mica/AA/HA/TiO_2_/DPPG were so pronounced that they exerted a measurable impact on the corresponding surface free energy parameters. These two surfaces exhibited markedly higher hydrophilicity (Figure 4) relative to the other tested samples; notably, their hydrophilicity was only marginally lower than that observed for the surface without a lipid film. This suggests that TiO_2_ particles likely reside directly beneath the lipid layer surface. The presence of positively charged TiO_2_ species promotes strong electrostatic interactions with the –PO_4_^−^ group of DPPG, thereby perturbing and potentially reorganizing the entire molecular fragment, including both the charged hydrophilic headgroups and the associated hydrophobic acyl chains.

**Figure 6:**
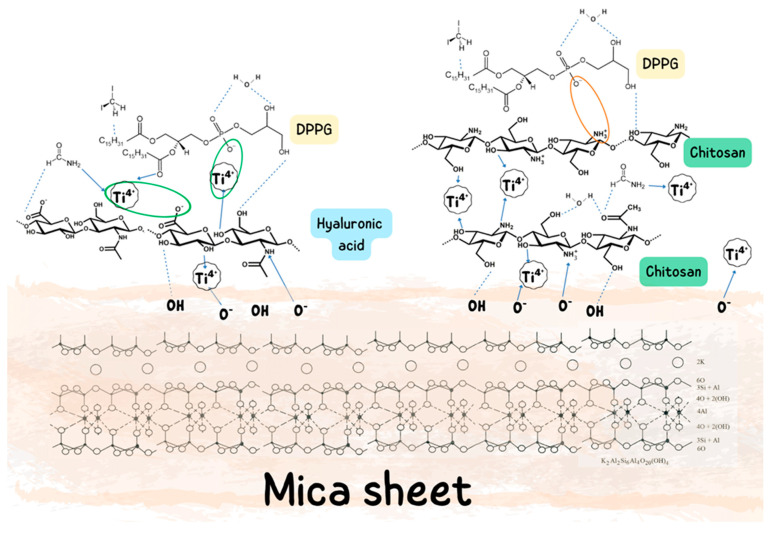
Schematic representation of the possible mechanism of interactions between Ch, HA, and nano-TiO_2_ with phospholipid DPPG on mica surface.

The interactions occurring between chitosan (Ch), hyaluronic acid (HA), TiO_2_ nanoparticles, and the DPPG lipid film determine both the molecular organization and the topography of the resulting model systems. In the Ch–DPPG system, a dominant role is played by strong electrostatic interactions between the protonated amino groups of chitosan (–NH_3_^+^) and the anionic phosphate of DPPG (–PO_4_^−^), further supported by hydrogen bonding (Figure 6). This leads to intensive biopolymer binding to the lipid surface and partial reorganization of the lipids. In contrast, HA–DPPG interactions are mainly hydrogen-bonding and hydrophilic in nature, accompanied by electrostatic repulsion resulting from the negative charge of both components which may led to the formation of a hydrated HA layer at the lipid film surface.

The introduction of TiO_2_ nanoparticles into the system leads to additional, strong donor–acceptor and hydrogen bond interactions. In the TiO_2_–DPPG system, the formation of Ti–O–P bonds and the binding of nanoparticles to the film surface can be observed, which results in the formation of nanodomains with a granular morphology. In contrast, in the Ch–TiO_2_ system, the nanoparticles are stabilized through coordination interactions with the –NH_2_ and –OH groups of chitosan, which promotes their uniform dispersity and limits agglomeration. In the case of HA–TiO_2_, the interactions are electrostatic and hydrogen-bonded in nature, but they lead to less ordered bonding, resulting in increased roughness and a tendency for the nanoparticles to aggregate.

Thus, a key role in shaping the surface properties of the modified DPPG films is played by electrostatic interactions between chitosan and lipids, as well as donor–acceptor and hydrogen interactions involving TiO_2_. These mechanisms initiate a reorganization of the lipid structure and lead to the formation of domains with diverse morphology and increased surface roughness. Finally, combining 2D and 3D characterization techniques, i.e., analyzing compression isotherms alongside the topography and hydrophilic–hydrophobic properties of monolayers transferred onto mica, provides a comprehensive overview of the interactions between the cell membrane model and its microenvironment.

## 4. Materials and Methods

### 4.1. Materials

To preparation the subphase, chitosan (Ch, Acrōs Organics, ACRS34905, Geel, Belgium) with a molecular weight of 100,000–300,000 (DD = 82 ± 2% [22]), commercial hyaluronic acid (high molecular weight, 1.60–1.80 MDa) in the form of a 1% sodium hyaluronate solution (HA, mazidla.com, Poznań, Poland), and TiO_2_ P-25 (Evonik, Essen, Germany, early Degussa) were used. SEM analysis revealed that the primary TiO_2_ particles, which readily aggregate, are approximately spherical in shape, and the main particle size fractions fall within the range of 10–30 nm, consistent with the manufacturer’s data for the commercial product (nominal particle size ~21 nm) [22,56]. Diluted acetic acid (AA, 99.9% purity) served as the solvent for these substances. To model a bacterial membrane, 1,2-dipalmitoyl-*sn*-glycero-3-phospho-*rac*-(1-glycerol) sodium salt (phospholipid DPPG ≥ 99%, Sigma-Aldrich, Steinheim, Germany) was dissolved at a concentration of 1 mg/mL in a 4:1 (*v*/*v*) mixture of chloroform (98.5%) and methanol (99.8%). Acetic acid, methanol, and chloroform were purchased from Avantor Performance Materials (Gliwice, Poland). Freshly cleaved mica sheets (60 × 50 × 0.2 mm^3^) from Continental Trade, Poland, were used for monolayer deposition. Contact angle measurements were performed using water purified by the Milli-Q water (Merck KGaA, Darmstadt, Germany) with a resistivity of 18.2 MΩ cm, as well as analytical-grade formamide and diiodomethane (Sigma-Aldrich, Steinheim, Germany).

### 4.2. Methods

#### 4.2.1. Langmuir–Blodgett Films Preparation

The transfer of the DPPG monolayer from the liquid–air interface onto a mica sheet was carried out using a thermostated Langmuir–Blodgett (LB) trough KSV 2000, KSV Instruments, Helsinki, Finland. The following subphases were utilized: 0.1% AA, Ch in AA 0.1 mg/mL, TiO_2_ in AA 1.2 mg/mL, HA in AA (*v*/*v* 0.5 mL/L) and their respective mixtures. The mica plate was placed in a vertical position and was pulled out from the subphase at a constant speed of 5 mm/min while maintaining a surface pressure of 35 mN/m. After deposition, the samples were stored in a vacuum oven prior to further analysis.

#### 4.2.2. Surface Topography

The most common characterization techniques used to analyze surface topography and height profiles of the deposited films are summarized in Table 2 [57,58,59,60,61,62,63].

The choice of a specific technique depends on the required resolution, the type of information sought (e.g., topography, mechanical properties, or thickness), the measurement conditions, and the nature and delicacy of Langmuir films.

Considering the advantages and disadvantages of the available techniques (Table 2), optical profilometry was determined to be the most suitable method for analyzing the DPPG monolayers transferred onto mica sheets from subphases containing Ch, HA and TiO_2_. Optical profilometry is a non-contact, non-destructive technique used to measure the surface topography and thickness of thin films. It provides high-resolution 3D surface maps, which are crucial for characterizing the topography of Langmuir–Blodgett films.

The substrate surfaces, onto which Langmuir monolayers were deposited from the respective subphases, were analyzed using an optical profilometer (Contour GT, Bruker, Karlsruhe, Germany).

#### 4.2.3. Contact Angle Measurements and Surface Free Energy Estimation

The contact angle measurements were conducted in a thermostatic chamber at a constant temperature of 20 °C, using a GBX apparatus (GBX Scientific, Romans-sur-Isère, France). This system was equipped with a camera to capture images of liquid drops on the sample surface and WinDrop++ software 4.1 to analyze the registered drop profiles. The drop volume of 6 μL was precisely deposited using a micropipette.

Based on the contact angle data, the total surface free energy and its components were calculated using the Lifshitz–van der Waals/acid–base (LWAB) approach. According to this model, the total surface free energy consists of the apolar Lifshitz–van der Waals component ($\gamma_{s}^{LW}$), which accounts for long-range interactions (dispersion, orientation, and induction), and the polar Lewis acid–base ($\gamma_{s}^{AB}$) component. The latter is further characterized by electron-donor ($\gamma_{s}^{-}$) and electron-acceptor ($\gamma_{s}^{+}$) parameters (1) [64,65,66,67].

(1) $$\gamma_{s}=\gamma_{s}^{LW}+\gamma_{s}^{AB}=\gamma_{s}^{LW}+\sqrt{2\left(\gamma_{s}^{+}\gamma_{s}^{-}\right)}$$

## 5. Conclusions

In this study, the DPPG monolayers were deposited from subphases containing chitosan, hyaluronic acid, and titanium dioxide onto mica sheets via the Langmuir–Blodgett technique, and their surfaces were characterized by optical profilometer, hydrophilic–hydrophobic character and wettability evaluation. Analysis of the energy-topographic parameters of the DPPG lipid surface, a model component of bacterial membranes, after contact with these biopolymers and TiO_2_ indirectly confirmed that the addition of TiO_2_ enhances antibacterial properties. The presence of nano-TiO_2_ is clearly manifested as a topographical irregularity and increased surface heterogeneity, which likely arise from partial incorporation or adsorption of TiO_2_ nanoparticles within or beneath the DPPG monolayer. Such structural perturbations can induce local rearrangements of the lipid headgroups and destabilize the monolayer structure. This modification of the lipid architecture is further reflected in the energetic parameters (total SFE and its components values). The resulting increase in surface roughness and energetic heterogeneity may facilitate the generation of reactive oxygen species (ROS) under ambient or photoactivated conditions, further contributing to the antibacterial effect. Therefore, the surface characterization of DPPG monolayers transferred onto mica is essential not only for understanding fundamental lipid–biopolymer–nanoparticle interactions but also for establishing the structure–function relationships that underpin antibacterial efficacy. Such insights are crucial for the rational design of next-generation biomaterials that combine controlled surface architecture with targeted antimicrobial functionality. We believe the presented research contributes to the field of molecular chemistry in several important ways, as detailed below.

Understanding of Molecular Interactions at Membrane Interfaces

This study provides detailed insights into how biological active molecules, chitosan, hyaluronic acid, and titanium dioxide nanoparticles, interact at the molecular level with model bacterial membranes. It elucidates changes in membrane physicochemical properties by correlating topographical features with compression isotherms and elasticity, thereby advancing knowledge of molecular interactions within complex biological interfaces.

Mechanistic Insights into Antibacterial Action

By analyzing the specific roles of each component and their potential synergistic effects, this research clarifies the molecular mechanisms behind membrane destabilization. This deepens the understanding of how biopolymers and nanoparticles disrupt bacterial membranes through electrostatic interactions and structural perturbations at the molecular level.

Design of Functional Molecular Mixtures

This study demonstrates how combining biologically active polymers (chitosan, hyaluronic acid) with inorganic nanomaterials (TiO_2_) can be rationally designed to modulate the molecular structure and function of membranes. This advances molecular chemistry by demonstrating how hybrid molecular systems can be constructed to achieve targeted biological activity.

Advancement in Surface Chemistry and Membrane Biophysics

The analysis of Langmuir films deposited on the solid supports, combined with compression isotherms and elasticity data, provides valuable molecular-level data on membrane biophysics. This enriches the fundamental understanding of lipid–biopolymer–nanoparticle interactions, which is crucial for designing new molecular materials with biomedical applications.

Potential for Translational Molecular Applications

The molecular insights gained pave the way for the development of novel antimicrobial materials and coatings, linking fundamental molecular chemistry with practical applications in medicine and biotechnology.

In summary, the molecular topography of Langmuir-Blodgett films is closely related to the lipid arrangement, which is influenced by hydrophilic and hydrophobic interactions, surface pressure during formation, and substrate properties. Controlling and modifying surface roughness is crucial in the design of effective antibacterial surfaces. The composites studied herein offer versatile and effective solutions for applications not only in medical devices and wound healing, particularly in healthcare, but also in food packaging and other areas where bacterial contamination is a primary concern. We hope that the research presented in this paper will bring us closer to achieving these goals, open up new directions of research, and facilitate further applications.

## Figures and Tables

**Table 1:** Topographical parameters characterizing the surface topography of DPPG monolayers transferred onto mica sheets from individual subphases. *R_a_*—arithmetical mean deviation; *R_q_*—root mean square roughness; and *R_t_*—total height of the profile.

Mica with Film	$R_{a}$ [nm]	$R_{q}$ [nm]	$R_{t}$ [nm]
H_2_O/DPPG	0.23 ± 0.05	0.27 ± 0.06	1.91 ± 0.32
AA/DPPG	0.26 ± 0.11	0.32 ± 0.13	2.32 ± 0.76
AA/Ch/DPPG	0.32 ± 0.14	0.27 ± 0.15	2.29 ± 0.63
AA/HA/DPPG	2.91 ± 1.01	3.43 ± 0.75	15.79 ± 3.25
AA/TiO_2_/DPPG	3.40 ± 2.32	4.22 ± 1.92	33.36 ± 8.47
AA/Ch/HA/DPPG	0.15 ± 0.07	0.18 ± 0.08	1.51 ± 0.36
AA/Ch/TiO_2_/DPPG	1.96 ± 0.55	2.80 ± 0.74	38.85 ± 3.61
AA/HA/TiO_2_/DPPG	4.93 ± 1.61	6.45 ± 1.66	73.83 ± 8.26
AA/Ch/HA/TiO_2_/DPPG	2.02 ± 0.99	2.70 ± 1.15	34.07 ± 3.65

**Table 2:** The advantages and disadvantages of the most commonly used techniques for topographical analysis of Langmuir films deposited on a solid substrate.

Technique	Advantages	Disadvantages
Atomic Force Microscopy (AFM)	- Very high spatial (nanometer) resolution [57,59,60]	- Time-consuming analysis [57,58]
	- Possibility of imaging under environmental conditions (air, liquid) [57,58,59,60]	- Potential damage to delicate films by the probe [57]
	- Information on the topography and mechanical properties of the surface [57,58,59,60]	- It requires specialized equipment and experience [57,58]
Scanning Electron Microscopy (SEM)	- Large depth of field and high resolution [57]	- The need for a vacuum and often for coating the sample with a conductive layer (e.g., gold) [57]
	- Fast surface analysis	- Possible thermal damage to delicate films [57]
	- Good imaging of surface morphology [57]	- No information about mechanical properties [57]
Optical Profilometry (OP)	- Non-invasive, fast, and contactless method [61,62]	- Limited vertical and horizontal resolution compared to AFM and SEM [62]
	- Possibility to measure thickness and topography over larger areas [61]	- Difficulties with measurements on highly rough or transparent surfaces [61,62]
Elipsometry	- Enables mapping the thickness and optical properties of films [63]	- Limited to thin, homogeneous films [63]
	- Fast and non-invasive [63]	- Lower spatial resolution [63]
Confocal Microscopy	- Possibility of 3D imaging of the surface and film layers [62]	- Limited lateral resolution compared to AFM and SEM [62]
	- Capability of imaging in humid and biological conditions [62]	- Requires fluorescent labeling or contrast difference [62]

## Data Availability

The original contributions presented in this study are included in the article. Further inquiries can be directed to the corresponding authors.

## Abbreviations

The following abbreviations are used in this manuscript:

Ch	Chitosan
HA	Hyaluronic acid
nano-TiO_2_	Titanium dioxide nanoparticles
DPPG	1,2-dipalmitoyl-*sn*-glycero-3-phospho-*rac*-(1-glycerol) sodium salt
LB	Langmuir–Blodgett technique
SFE	Surface free energy
